# Remote Therapeutic Monitoring in Rheumatic and Musculoskeletal Diseases: Opportunities and Implementation

**DOI:** 10.18103/mra.v11i7.2.3957

**Published:** 2023-07

**Authors:** W. Benjamin Nowell, Jeffrey R. Curtis

**Affiliations:** 1 Global Healthy Living Foundation, Upper Nyack, NY, USA; 2 Illumination Health, Hoover, AL, USA; 3 University of Alabama at Birmingham, Birmingham, AL, USA

**Keywords:** Rheumatoid Arthritis, Psoriatic Arthritis, Ankylosing Spondylitis, Telemedicine, Patient-Reported Outcomes

## Abstract

Therapeutic Monitoring (RTM) is a new program in the United States that began in 2022 allowing electronic patient-reported outcomes (ePRO) and other patient-generated data to be reviewed by clinical staff between visits so that patients can receive clinical attention as needed. Remote Therapeutic Monitoring simultaneously enhances the capacity to generate prospective longitudinal data that may be useful for secondary research purposes. As many governmental and private insurance programs in the United States now provide reimbursement for Remote Therapeutic Monitoring, increasing numbers of rheumatologists may be incentivized to provide this service for their patient populations. Launched in 2015, the ArthritisPower^®^ Research Registry and associated mobile and desktop application, registered with the Food & Drug Administration (FDA) as a Class I medical device, enables patients to track their disease across dozens of domains and to securely participate in voluntary research studies. ArthritisPower, in partnership with Illumination Health, has developed infrastructure and a clinical workflow for Remote Therapeutic Monitoring that will help rheumatologists more closely track their patients’ disease activity and flares, identify primary non-adherence, record changes in key health domains (e.g. fatigue, pain, physical function, mental health) and meet the needs for other data elements important for clinical care identified by individual providers. Ultimately, the approach to use digital health tools between visits seeks to improve clinical outcomes for patients with rheumatic and musculoskeletal diseases. This editorial review discusses the evolution of remote monitoring in rheumatologic care, describes the opportunities for physician reimbursement as of 2023, and provides a suggested workflow in order to establish Remote Therapeutic Monitoring within rheumatology practices.

## History and Background

Remote patient monitoring is a technology-based health care delivery system that allows health care providers to remotely track patients’ health status using various digital devices. It has the potential to improve patient outcomes by allowing health care providers to monitor patients in real-time, detect problems early, and adjust treatment plans accordingly. It can also reduce the need for in-person visits and unscheduled visits (e.g. to the emergency department), saving patients time and reducing health care costs.^1^ There are two general types of remote patient monitoring: Remote Physiologic Monitoring (RPM) and Remote Therapeutic Monitoring (RTM). Remote Therapeutic Monitoring refers to the use of app-based technology to monitor and manage patients, particularly those with chronic conditions such as rheumatoid arthritis. While Remote Physiologic Monitoring focuses on monitoring vital signs, biometrics, and symptoms using physiologic sensor devices (with or without an accompanying smartphone app), Remote Therapeutic Monitoring requires only a smartphone app and focuses on monitoring medication adherence, adverse reactions, and therapeutic outcomes.

In orthopedic care, Remote Physiologic Monitoring has been used to monitor patients with musculoskeletal disorders before and after surgery such as joint replacement (arthroplasty).^2^ In this context, patients can use Remote Physiologic Monitoring devices to record and transmit data on aspects of their health such as pain, range of motion, and physical activity levels. Peri-operative management considerations that are amenable to measurement with a biosensor can be useful to help confirm that the patient has been well optimized medically with respect to blood pressure, healthy body weight, etc^3^. Health care providers can use Remote Therapeutic Monitoring and Remote Physiologic Monitoring data to monitor the patient’s progress, adjust therapeutic regimen, provide feedback and support, and gauge when specific interventions (e.g. surgery) are most opportune.^4^

While Remote Physiologic Monitoring has been used for many years in routine care for patients with diabetes (with continuous glucose monitoring), hypertension (with home ambulatory blood pressure monitoring), and heart failure (e.g. via Wi-Fi-equipped scale), its widespread adoption and acceptance as a reimbursable service by the United States Centers for Medicare & Medicaid Services (CMS) is a more recent development. Additionally, CMS has recently expanded reimbursement to include Remote Therapeutic Monitoring. The beginning of the inclusion of these technology services was in 2018 when CMS released the “Final Rule” which allowed Remote Physiologic Monitoring services to be reimbursed by Medicare beginning in 2019. The associated Current Procedural Terminology (CPT) codes apply to patients with chronic conditions where a physiologic biosensor device was useful, including hypertension, diabetes, and heart failure. The insurance reimbursement included coverage for both the setup, data transmission, and monthly time spent monitoring the data and reacting accordingly, e.g. adjusting medication regimens. In 2022, CMS added five new Current Procedural Terminology codes for Remote Therapeutic Monitoring to the Medicare Physician Fee Schedule in a parallel fashion to the corresponding Remote Physiologic Monitoring codes (see Table 1). Like the Remote Physiologic Monitoring codes, Remote Therapeutic Monitoring allows for reimbursement for setup (CPT 98975) and data transmission (CPT 98976, 98977), as well as the two time-based codes, CPT 98980 and CPT 98981, in increments of 20 minutes per month. Remote therapeutic monitoring is specific to patients with musculoskeletal and respiratory conditions using a digital app [i.e., Software as a Medical Device (SaMD)] and does not require a physiologic biosensor device. These programs have the potential to improve patient outcomes by increasing medication adherence and reducing the risk of adverse drug events as any disease activity or clinically relevant information is now reimbursable.^5^ The use of Remote Physiologic Monitoring has exploded as digital data collection between visits (via digital care pathways, implemented by software apps and enriched by data streams from those apps and/or physiologic biosensor devices) became reimbursable by insurance. While initially fee for service Medicare led the way, a growing number of other insurance plans, both governmental (e.g. Medicaid) and commercial, now cover Remote Physiologic Monitoring and Remote Therapeutic Monitoring^6,7^. The evolution of digital medicine has played a large role in the extension of reimbursement for telehealth-related services, including those for Remote Physiologic Monitoring.^8^ A recent analysis that used Medicare monthly claims volume to measure uptake of remote monitoring use reported a 555 percent increase in use from February 2020 to September 2021^9,10^. From the inception of the program in 2019 to the most recent Medicare data available in December of 2021, Remote Physiologic Monitoring has grown 19-fold ^11^.

The COVID-19 pandemic also accelerated adoption of remote monitoring, and of telehealth services, generally. This was fueled by patients’ desire for virtual visits to reduce their risk for SARS-Cov-2 infection, increased interest by providers to offer telehealth to maintain patient contact while minimizing in-person visits, and regulatory changes implemented by the COVID-19 Public Health Emergency (PHE) that allowed for reimbursement of telehealth and a variety of other remote services^13^. According to a recent McKinsey and Company report, rheumatology is among the top five specialties to engage in telehealth ^14^. McKinsey’s report also “shows between 40 and 60 percent of consumers express interest in a set of broader virtual health solutions, such as a ‘digital front door’ or a lower-cost virtual-first health plan.” In rheumatology, a recent study we conducted found that 60 percent of rheumatology patients prefer telemedicine for routine visits and review of their laboratory results^15^. Additionally, a systematic review of studies from August 2015 to January 2022 found telemedicine to be an effective way of delivering care for those with rheumatic disease, matching outcomes in usual/in person care.^16^ In this context, remote physiologic and Remote Therapeutic Monitoring may serve as a useful adjunct to telehealth visits, helping fill in the gaps as to how patients are doing between in-person or telehealth visits and lessen provider’s concerns that they might be missing an important change in their patients’ health status. Providers also benefit by being able to schedule an earlier visit, maintain a patient’s progress in their health journey, and help patients to stay accountable for their own health with assistance from the provider’s office.

### Remote Therapeutic Monitoring

For people living with chronic, rheumatic disease who need ongoing care, the increased availability of telemedicine and Remote Therapeutic Monitoring during the pandemic was a boon, considering this patient population’s heightened risk of infection and associated serious complications. However, despite more patients engaging in telehealth than ever before,^17,18^ the American College of Rheumatology reported in a 2020 survey that Americans living with a rheumatic disease still face significant challenges to access care. It remains to be seen how the end of the COVID-19 Public Health Emergency in the United States, as of May 11, 2023, will ultimately affect the availability of telehealth for patients, given that not all telehealth Current Procedural Terminology codes were made permanent. While Remote Physiologic Monitoring and Remote Therapeutic Monitoring are expected to continue for the foreseeable future, patient copayments were waived during the Public Health Emergency. However, after May 2023 when the COVID-19 Public Health Emergency ended, patients with insurance plans that required a monthly copayment (e.g. $5-$20/month) became responsible for this additional expense, highlighting the importance of demonstrating the value of Remote Therapeutic Monitoring program to the patient. Further, if Remote Therapeutic Monitoring is to expand in rheumatology practices across the country to make it a standard practice, a number of technological and reimbursement considerations must be worked out. For example, it will be necessary to ensure that patients have equitable access to a smartphone with adequate Wi-Fi or cellular coverage to complete assessments remotely. Early studies of electronic patient-reported outcomes in cancer care have suggested that racial and ethnic minorities, older patients, and those with less education may actually benefit even more from digital patient-reported outcomes if implemented properly.^19^ Without remote monitoring, such patients may not be getting the medical attention they need because of resource restrictions, prohibitive distance to travel to their in-office provider appointments, or the inability to convey symptoms without a language translator. Demonstrating and emphasizing the cost saving component of Remote Therapeutic Monitoring to private insurance carriers will also be instrumental.

### Rationale for Remote Therapeutic Monitoring in Rheumatology

Remote therapeutic monitoring entails patients regularly providing information such as patient-reported outcomes, their experience of symptoms and disease activity, satisfaction with care, and medication uses and perceptions. These data are sent to a health care provider and their staff for regular monitoring and evaluation. But why should rheumatology providers, in particular, wish to have patient data between visits?

First, such information can complement telehealth visits. Telehealth is vital in rheumatology because patients living with chronic conditions require ongoing check-ups on their disease activity, symptoms, and treatment. While it is not necessary for patients to be seen in person by a provider for each visit, Remote Therapeutic Monitoring may assist clinical evaluation, potentially lowering the amount of time required for a telehealth visit, since patients have shared validated patient-reported outcomes for disease activity information such as number and length of flares, self-joint count, or other patient-reported outcome measures (pain, physical function, sleep) using an FDA-registered software as a medical device. For example, patients could use Remote Therapeutic Monitoring to spend 5 minutes per week providing patient-reported outcome information between visits, greatly increasing confidence that a seemingly stable patient is doing well with ample longitudinal data. Patients may be especially motivated to provide Remote Therapeutic Monitoring data between visits, over time, if it means having a telehealth appointment in lieu of an in-person visit. This is because telehealth visits tend to reduce the time, effort and money required of patients. Patients seek health care that is convenient and that enables quality connections with providers; the explosion of telehealth technologies has allowed for this while enabling patients to be better involved in their own care.^20^ Our recent ArthritisPower study showed that patients with autoimmune rheumatic diseases frequently had telemedicine visits during the COVID-19 pandemic, with the majority held via video, and that patients were satisfied with these visits ^15^. In particular, patients preferred telehealth (video or phone) visits for delivery of routine visits, such as reviewing lab results.

Second, Remote Therapeutic Monitoring may improve rheumatologists’ patient care. Conditions such as rheumatoid arthritis, ankylosing spondylitis, psoriatic arthritis, and lupus are unpredictable, and disease flares are common. With only scheduled follow-up visits to gather information, conventional clinical practice risks abandoning patients to unsupervised approaches to dealing with their flares, pain management, and medications between appointments. While many patients are likely to contact their provider’s office when they need consultation, others may not, and important opportunities to change treatment to effect better disease control may be missed in the absence of close monitoring. Remote therapeutic monitoring technology that is built into the clinical workflow has great potential to improve clinical outcomes. By helping patients submit indicators of their disease activity to providers as often as daily or weekly, Remote Therapeutic Monitoring enables rapid detection of changes in a patient’s health. In addition, by facilitating a monthly phone call between a patient and their provider’s office, Remote Therapeutic Monitoring allows for quick follow up on health concerns that the patient or provider may have. A recent randomized controlled trial conducted in the Netherlands implementing a digital health program similar to Remote Therapeutic Monitoring in stable rheumatoid arthritis patients showed that patients’ disease activity remained under good control (adjusted difference in DAS28 −0.04 units, 95% CI −0.39 − 0.30), was associated with a lower number of disease flares, and significantly reduced the number of rheumatologist and nurse consultations ^21^. Remote patient monitoring devices for those with chronic conditions reduces mortality and improves disease activity as indicated by blood pressure and glycated hemoglobin, for example.^22^ Although other types of health care utilization were not studied, a recent systematic review found that remote monitoring reduced hospitalizations among chronic obstructive pulmonary disease and cardiac patients ^23^.

Third, Remote Therapeutic Monitoring can enable data collection to satisfy quality reporting through the Merit-based Incentive Payment System (MIPS). In the United States, this system is used in rheumatology to incentivize eligible clinicians to provide high-quality, cost-effective care to patients. Eligible clinicians are scored on four performance categories and are eligible for financial rewards or penalties based on their score. Several Merit-based Incentive Payment System measures are directly reportable using Remote Therapeutic Monitoring solutions. For example, patients can report on domain specific patient-reported outcomes such as depression (MIPS Measure 134) and disease specific patient-reported outcomes (e.g. MIPS Measure 177, Disease Activity Assessment in RA; MIPS Measure 178, Functional Status Assessment in RA).

Finally, as a secondary benefit to its implementation for clinical care, Remote Therapeutic Monitoring has the potential to support research by enhancing study data capture between visits with the capacity to generate comprehensive prospective longitudinal data. Specifically, it allows patient-reported outcomes captured at regular intervals to be paired with patient-reported medication starts and stops, or with biosensor, actigraphy data, or with clinical data from provider visits to create or complement a disease registry. Moreover, Remote Therapeutic Monitoring can improve screening for clinical trials by flagging which individuals are most likely to be eligible or interested in a particular study where such screening benefits from information directly from patients.

### Remote Therapeutic Monitoring/Remote Physiologic Monitoring Implementation Evidence is Lacking

Despite the strong rationale for its use and a promising new reimbursement structure, there is yet little evidence in rheumatology for Remote Therapeutic Monitoring. Information about best practices for implementation and the impact of Remote Therapeutic Monitoring on patient outcomes is sorely needed in order to provide an evidence base that can inform the utilization of this technology. Although not entirely specific to Remote Therapeutic Monitoring-like interventions, a recently published, systematic, meta-analysis that informed a task force that led to the 2022 EULAR Points to Consider for remote care in rheumatic and musculoskeletal diseases found that remote care leads to similar or better results compared with face-to-face treatment specific to efficacy, safety, adherence and user perception outcomes^24^. In this study, “the most frequently studied intervention was remote monitoring (i.e., telehealth-based monitoring of disease activity or function) (n=35; 74%), followed by remote diagnostics (n=2; 4%). Remote care was mostly delivered using telephone/video calls (n=30; 64%), and in 10 studies, all of them [randomized controlled trials], an individual edevice was used for data collection (21%).” As the authors explain, the technical aspects of remote care were considered both drivers and as barriers to satisfaction. Providers will need to bolster technology literacy among certain patients, and patients will need to feel confident in how using the technology is a benefit to them, in terms of its potential to improve health outcomes. The authors rightly conclude that more study of Remote Therapeutic Monitoring is needed, particularly with the advancement of technologies and usage during and after the COVID-19 pandemic.

Early research to better understand whether people living with rheumatic disease will participate in Remote Therapeutic Monitoring, and whether it is helpful to improve outcomes, is encouraging. In a study of rheumatoid arthritis, patients invited to daily provide patient-generated health data using smartphones and then integrating that data into the electronic health record, researchers reported that the “system helped render patients’ rheumatoid arthritis more visible by providing the ‘bigger picture’, identifying real-time changes in disease activity and capturing symptoms that would otherwise have been missed.”^25^ Recent studies involving passive data collection (using smart watches, smart phones and other fitness devices) in rheumatoid arthritis suggest remote tracking is feasible but that programs benefit from patient-centered implementation and design to minimize patient burden, promote longitudinal engagement and maximize adherence^26^. Adherence is key, as Remote Therapeutic Monitoring is a more active form of tracking, requiring providers and their staff to promote its ongoing use while analyzing the incoming data and patients to commit to participation. Furthermore, Remote Therapeutic Monitoring is useful only inasmuch as it is able to inform future patient care or contribute to real-world data about a condition and its treatment^27,28^. A recently published systematic literature review of the clinical impact of electronic patient reported outcome monitoring summarized 8 studies (7 of them in rheumatoid arthritis) as a digital intervention and showed that monitoring had a small impact on improving disease activity (standardized mean difference −0.15, 95% CI −0.27, −0.03) and more substantial impact on decreasing health care utilization through more effective triage (standardized mean difference −0.3, 95% CI −2.14, 0.28).[ref]

### The Signal and Bridge to Connected Care: Using the ArthritisPower Patient App to Implement Remote Therapeutic Monitoring

Simply explained, remote monitoring requires the following general steps or components: (1) the doctor/care team recommends Remote Therapeutic Monitoring program to the patient; (2) the patient signs up (registers) for the Remote Therapeutic Monitoring program and downloads/sets up software to provide data; (3) the health care team (i.e. office staff) calls the patient to welcome the patient and address any questions the patient may have about the program, since they first learned about the program; (4) the patient regularly completes assessments and provides other data; (5) the health care team reviews the patient data for at least 20 minutes per month to monitor for significant out-of-range values in patient data, calls the patient to follow up on these, reminds the patient to provide data regularly and remain adherent with the program, call the patient once a month to help capture any health concerns the patient may have aside from their symptoms reported remotely (such as inability to start their newly prescribed medication due to financial reasons); and (6) the health care team escalates any worsening symptoms or patient concerns to the provider, if the need arises, and follows the subsequent orders given in this regard such as scheduling an earlier visit or sending a new prescription.

#### ArthritisPower App as an example of a Remote Therapeutic Monitoring Solution

From the patient perspective, there are an overwhelming number of mobile applications to download that purport to provide a comprehensive experience for individuals living with arthritis to track their symptoms,^29^ but few of these apps use validated measures that are captured securely. Launched in 2015, the ArthritisPower^®^ Research Registry is a patient-centered research registry for joint, bone, and inflammatory skin conditions, as well as arthritis and rheumatologic manifestations of gastrointestinal-tract and skin conditions ^30–32^. The patient-facing mobile application created by the ArthritisPower registry and registered with the United States Food and Drug Administration (FDA) as a Class I medical device can serve as software as a medical device for Remote Therapeutic Monitoring and facilitate the collection of validated patient-reported outcome measures which allows for continuous disease and symptom monitoring by health providers.

**Figure 1.**
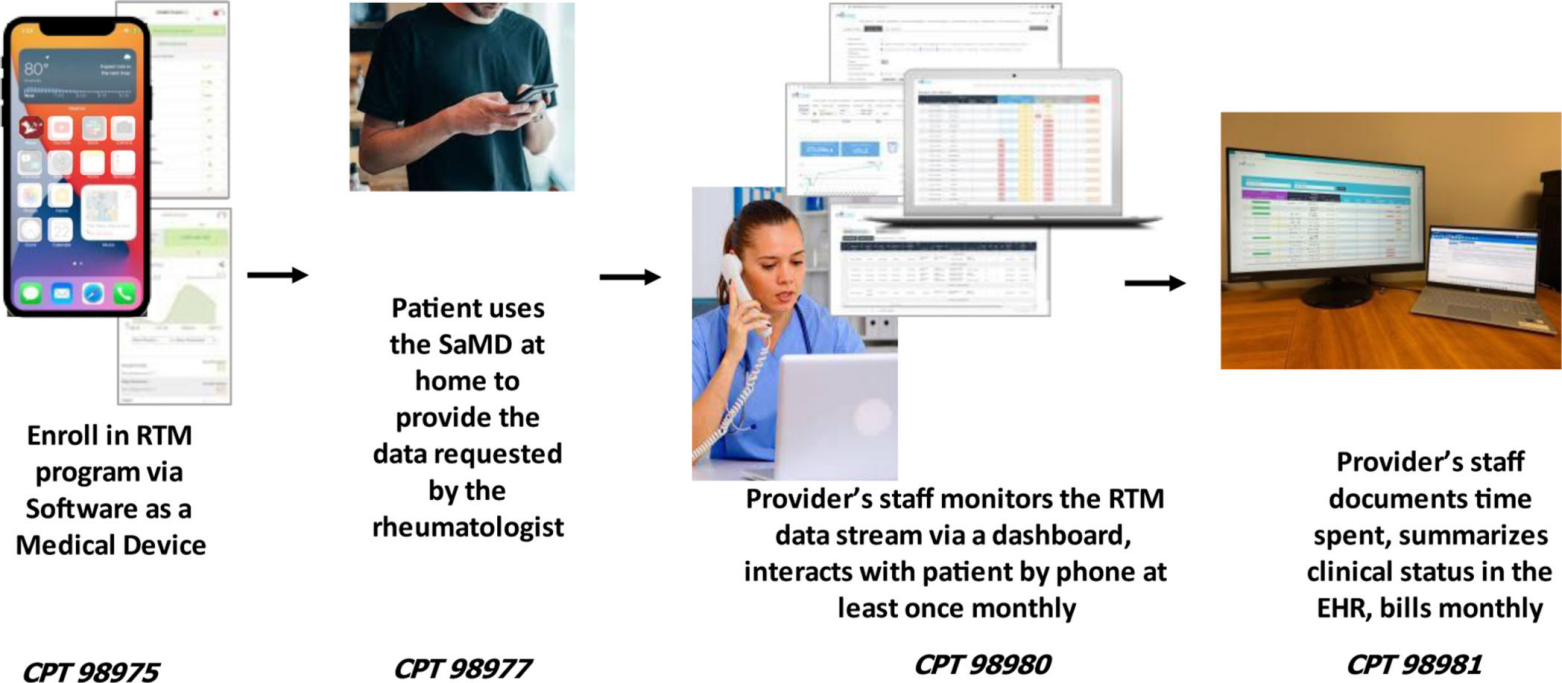
Remote Therapeutic Monitoring Components RTM=Remote Therepeutic Monitoring; SaMD= Software as a Medical Device; CPT = Current Procedural Terminology; EHR = Electronic Health Record

The process by which Remote Therapeutic Monitoring is deployed is shown in Figure 1. During an appointment, a provider will introduce Remote Therapeutic Monitoring as part of a comprehensive patient disease management strategy, not unlike how medicines are prescribed, taken at a specific frequency on a defined timeline and covered by insurance, and the patient is enrolled. In conjunction with patients, one of several specific remote monitoring pathways is selected, and the software as a medical device then is deployed to track the key symptoms embedded in that monitoring pathway using the ArthritisPower app. Patients’ data will flow into a patient dashboard and subsequently to the electronic health record (EHR) where it will be monitored at least 20 minutes per month by the rheumatologists’ office staff (e.g. medical assistant). While there is a connection between the patient and their health provider’s office, ArthritisPower complements but does not supplant a patient portal. The office staff completes the monthly billing for health insurance for Remote Therapeutic Monitoring.

**Figure 2.**
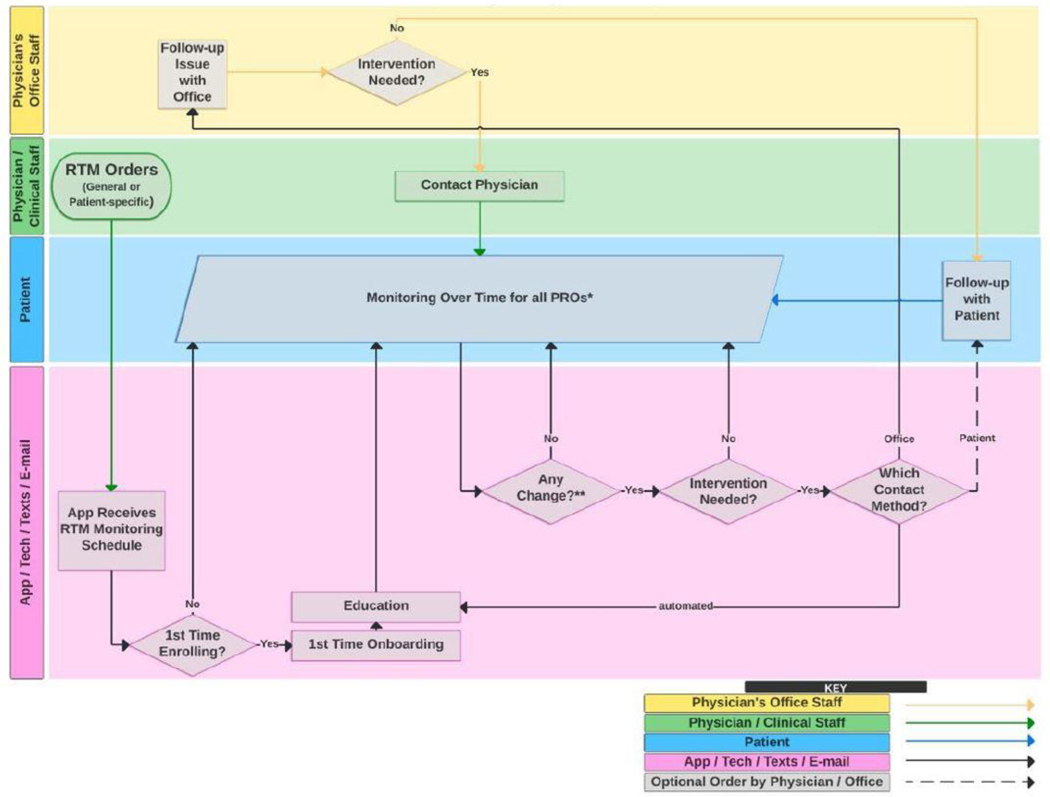
Remote Therapeutic Monitoring Workflow

One example of a remote monitoring pathway and its associated data flow and actions is shown in Figure 2 and describes which actions are taken by which agents (e.g. patient, provider, office staff, and the technology). Its more detailed specification (not shown) provides information about the type of data (i.e. the patient-reported outcome measures and other instruments) to be collected, frequency and schedule for data collection by the app, and threshold for intervention. Different monitoring pathways can be deployed within each disease state, since the type and frequency of patient data may differ according to the disease state and the clinical scenario (e.g. medication start; medication tapering with disease activity monitoring; symptom management after health care services such as vaccination).

Researchers at the Global Healthy Living Foundation and Illumination Health are currently conducting an implementation study of RTM deployment in United States rheumatology care sites of more than 300 patients, with the intent to subsequently scale nationally. We are assessing patient and provider uptake, acceptability and patient adherence to remote monitoring care pathways. Patients treated by community rheumatology providers who are members of the Excellence Network in RheumatoloGY (ENRGY), a practice-based network (PBN) instantiated in 2022 (https://illumination.health/physiciannetwork/) are invited to participate. Patients will be on one of the customized RTM pathways specific to the relevant disease states (e.g. rheumatoid arthritis, spondyloarthritis, gout). This enrollment will be a part of the standard clinical workflow at the site, either before, during or immediately following a routine clinic visit/intake, and the proposed program will provide the data necessary for program evaluation.

## Conclusion

The increasing availability of technology-based tools to deliver health care outside of the medical office or hospital setting will benefit both patients and providers alike. For patients, at-home technology to aid in the delivery of telehealth and Remote Therapeutic Monitoring may help patients more proactively manage their disease according to their provider’s recommendations, particularly since it removes barriers to care such as transportation limitations, work schedule conflicts, and/or childcare disruptions^33^. Providers benefit from getting real-time data about their chronic disease patients, undiluted by faulty memory when assessed at in person visits appointments which typically occur every 2–6 months. As suggested by clinical trials, Remote Therapeutic Monitoring may also free up vital resources, such as clinical time in office, allowing providers to see more ill patients while stable patients remain remotely monitored^34^. Though potentially additive to the work performed by office staff, the fact that Remote Therapeutic Monitoring is now reimbursable is likely to incentivize additional uptake, while the true amount of incremental work needs to be quantified. For some patients, knowing that they will receive a monthly call by their office staff to check on them helps such patients save any minor questions or concerns for this time, thus redirecting the office workflow to this program.

Additional study is required to determine if and how Remote Therapeutic Monitoring can improve health outcomes, particularly among patient populations which may be less comfortable with or have reduced access to smartphone or desktop technologies, or other medical devices. There may be a steep technology learning curve as providers determine best practices for integrating Remote Therapeutic Monitoring in their workflow and create a practical hybrid model combining in-person visits with telehealth and Remote Therapeutic Monitoring solutions^14^. It will also be important that communities impacted by health disparities and/or digital literacy are not left behind as new technologies like Remote Therapeutic Monitoring are embraced. This may include older adults, communities of color or those for whom English is not their primary language at home, or people living in rural areas who could potentially benefit from telehealth and Remote Therapeutic Monitoring the most if their proximity to quality health care is limited by their location. Remote Therapeutic Monitoring and remote physiologic programs also need to clarify which patients will benefit the most and for what use cases. It might be patients with more severe or unstable disease with the greatest need for provider contact, although it could be stable patients for whom Remote Therapeutic Monitoring could be used to effectively triage more extended follow-up intervals because they are doing well. Education programs need to be created and put in place to ensure that patients feel confident in implementing their prescribed Remote Therapeutic Monitoring at home. In light of these considerations, Remote Therapeutic Monitoring promises to enable rheumatologists to more easily observe their patients in real time. Primary non-adherence, medication-related adverse events, and fluctuations in disease activity, flare, and other key health domains comprise critical information needed for health care decision making. The use of digital health tools between visits, such as smartphone apps to convey patient-generated data to provider practices, is soon to become both commonplace and indispensable to high quality care.

## Figures and Tables

**Table 1.** Current Procedural Terminology (CPT) Remote Physiologic and Remote Therapeutic Monitoring Codes^12^

CPT Codes (RPM, RTM)*	Example Description	Reimbursement (estimated)
99453 ***98975***	Initial set-up and patient education on use of equipment for remote monitoring of physiologic parameter(s) using a biosensor, and/or Software as a Medical Device (e.g. weight, blood pressure, pulse oximetry, respiratory flow rate)	$17 (one time)
99454 ***98977 (MSK)*** ***98976 (RSP)***	Device(s) supply with daily recording(s) or programmed alerts transmission, each 30 days.	$50 (every month)
99457 ***98980***	Remote Physiologic Monitoring treatment management services, clinical staff/physician/other qualified health care professional time in a calendar month, requiring interactive communication with the patient/caregiver 3nth; first 20 minutes.	$47 (every month)
99458 ***98981***	Each additional 20 minutes (max 60 minutes total)	$38 (up to 2/month)

CPT = Current Procedural Terminology; RPM = Remote Physiologic Monitoring;

RTM = Remote Therapeutic Monitoring

* Current Procedural Terminology (CPT) codes for specific types of monitoring also can be reimbursed, including CPT 96127, CPT G3002 and CPT G3003

Bold indicates new Current Procedural Terminology codes that were introduced in 2022 specific to RTM for patients with musculoskeletal (MSK) and respiratory (RSP) conditions
